# Detection of Pancreatic Cancer via Specific Metabolite Markers: A Metabolomics Approach

**DOI:** 10.1002/hsr2.73133

**Published:** 2026-08-26

**Authors:** Mohammad Javad Roustaye Gourabi, Mehrangiz Ghafari, Anita Nikoo, Yalda Yarahmadi Saki, Bita Khanbabaei, Masoumeh Khanizadeh, Masoud Kargar, Ali Hashemi, Javad Yasbolaghi Sharahi

**Affiliations:** ^1^ Department of Microbiology School of Medicine, Shahid Beheshti University of Medical Sciences Tehran Iran; ^2^ Department of Pathology School of Medicine, Zabol University of Medical Sciences Zabol Iran; ^3^ Department of Medical Parasitology and Entomology Tarbiat Modares University Tehran Iran; ^4^ Department of Biological Sciences and Technologies Faculty of Basic Sciences, Islamic Azad University, Central Tehran Branch Tehran Iran; ^5^ Department of Biological Sciences Roudehen Center, Islamic Azad University Roudehen Iran; ^6^ Department of Medical Microbiology (Bacteriology and Virology) Afzalipour School of Medicine, Kerman University of Medical Sciences Kerman Iran; ^7^ Thalassemia and Hemoglobinopathy Research Center Research Institute of Health, Ahvaz Jundishapur University of Medical Sciences Ahvaz Iran; ^8^ Student Research Committee, (Department of Microbiology) School of Medicine, Shahid Beheshti University of Medical Sciences Tehran Iran

**Keywords:** cancer detection, metabolic reprogramming, NMR spectroscopy, pancreatic cancer, pancreatic cancer metabolomics, PDAC biomarkers

## Abstract

**Background and Aims:**

Pancreatic ductal adenocarcinoma (PDAC) remains one of the most lethal malignancies worldwide because most patients are diagnosed at advanced stages when curative treatment is no longer feasible. Metabolomics has emerged as a promising strategy for identifying biochemical alterations associated with early tumor development and may improve the early detection of PDAC. This review critically evaluates the role of metabolomics in PDAC diagnosis, focusing on metabolite biomarkers, nuclear magnetic resonance (NMR) spectroscopy, mass spectrometry‐based platforms, and machine learning assisted approaches.

**Methods:**

A narrative review of the current literature was conducted to assess the diagnostic potential, analytical methodologies, and translational challenges of metabolomics in PDAC. Evidence related to metabolite biomarkers, analytical platforms, artificial intelligence applications, and clinical implementation was critically examined.

**Results:**

PDAC is characterized by significant alterations in glycolysis, amino acid metabolism, lipid metabolism, and microbiome derived metabolites. Several metabolite panels have demonstrated promising diagnostic performance, particularly when combined with CA19‐9. Advanced analytical platforms, including NMR spectroscopy, liquid chromatography mass spectrometry, and gas chromatography mass spectrometry, have enabled detailed characterization of metabolic signatures and improved discrimination between PDAC and benign pancreatic diseases such as chronic pancreatitis. However, clinical translation remains limited by methodological heterogeneity, biological variability, lack of standardized analytical workflows, interlaboratory reproducibility concerns, and insufficient prospective multicenter validation. Although machine learning has enhanced biomarker discovery and pattern recognition, challenges related to overfitting, interpretability, and external validation remain unresolved.

**Conclusion:**

Metabolomics is unlikely to replace existing diagnostic modalities in the near future but may substantially improve diagnostic accuracy when integrated with established biomarkers, imaging techniques, and molecular diagnostics. Future progress will depend on standardized protocols, large prospective validation studies, and clearly defined regulatory pathways. With these advances, metabolomics may become an important component of precision diagnostic strategies for PDAC.

## 1 Introduction

Pancreatic ductal adenocarcinoma (PDAC) remains one of the deadliest human malignancies, with mortality rates closely paralleling incidence rates because most patients are diagnosed at advanced stages of disease [1]. More than 80% of patients present with unresectable or metastatic tumors, resulting in a 5‐year survival rate that rarely exceeds 10% despite advances in surgery, chemotherapy, and supportive care [1]. The poor prognosis of PDAC is largely attributable to the absence of reliable strategies for detecting disease at an early and potentially curable stage. Consequently, improving early diagnosis has become a major priority in pancreatic cancer research and clinical practice. Recent studies have further emphasized that meaningful improvements in patient outcomes will depend on identifying biologically informative biomarkers capable of detecting PDAC before the onset of advanced local invasion or distant metastasis [2, 3].

Current diagnostic approaches remain imperfect. Although imaging modalities such as computed tomography, magnetic resonance imaging, and endoscopic ultrasound play essential roles in disease detection and staging, their sensitivity for identifying very early lesions remains limited. Similarly, the widely used serum biomarker CA19‐9 suffers from suboptimal sensitivity and specificity, particularly in early‐stage disease and inflammatory pancreatic conditions. Emerging molecular approaches, including circulating tumor DNA (ctDNA) analysis and liquid biopsy technologies, offer promising alternatives but continue to face challenges related to sensitivity, cost, and standardization. Within this context, metabolomics has emerged as a potentially valuable complementary strategy capable of capturing biochemical alterations that occur during the earliest phases of tumor development [4, 5].

**Figure 1:**
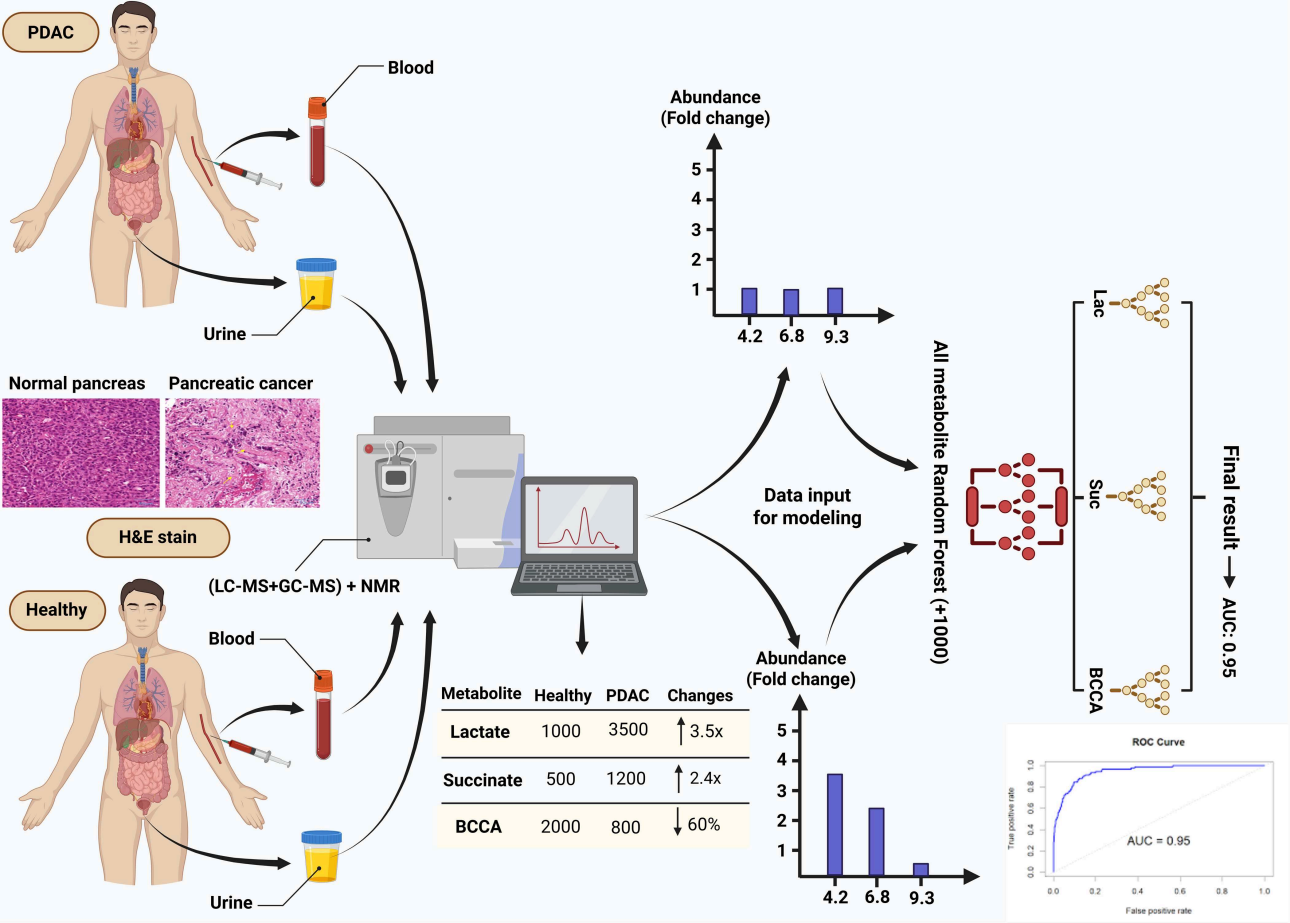
Schematic workflow of metabolomics‐based biomarker discovery and machine learning‐assisted classification in PDAC. Blood and urine samples from healthy individuals and PDAC patients are analyzed using LC‐MS, GC‐MS, and NMR platforms to identify metabolic alterations. Representative metabolites, including lactate, succinate, and branched‐chain amino acids (BCAAs), are shown as examples of metabolites that may increase or decrease in PDAC. The numerical values and fold‐change illustrations are schematic and intended to demonstrate relative metabolic changes rather than actual concentrations from a specific dataset. The resulting metabolomic profiles are integrated into machine learning models for biomarker selection and disease classification.

Metabolomics provides a comprehensive assessment of low molecular weight metabolites that reflect the functional state of cellular processes. In PDAC, extensive metabolic reprogramming involving enhanced glycolysis, altered amino acid utilization, glutamine dependency, lipid remodeling, and interactions with the gut microbiome has been consistently observed [6, 7]. These alterations have generated considerable interest in the development of metabolite‐based biomarkers for early detection, disease stratification, and therapeutic monitoring. As illustrated in Figure 1, integrated metabolomic profiling utilizes multiple analytical platforms to characterize these metabolic perturbations and potentially overcome some of the limitations associated with single biomarker approaches.

Despite its promise, the clinical translation of metabolomics remains far from straightforward. The field is characterized by substantial methodological heterogeneity, including differences in analytical platforms, sample preparation protocols, preprocessing workflows, and statistical methodologies. These variations contribute to inconsistent biomarker identification across studies and complicate comparisons between independent cohorts. Furthermore, biological variability arising from dietary habits, medication use, comorbidities, microbiome composition, and population specific factors presents additional challenges for biomarker reproducibility and clinical implementation.

Among available metabolomic technologies, nuclear magnetic resonance (NMR) spectroscopy has attracted particular attention because of its high reproducibility, minimal sample preparation requirements, and non‐destructive analytical capabilities [8, 9]. NMR enables simultaneous quantification of multiple metabolites and has demonstrated potential for differentiating PDAC from benign pancreatic diseases such as chronic pancreatitis. However, its theoretical advantages must be balanced against important limitations, including lower analytical sensitivity compared with mass spectrometry (MS), substantial infrastructure requirements, high operational costs, and limited evidence from large multicenter validation studies. Notably, despite decades of research, no NMR based diagnostic test has received regulatory approval for pancreatic cancer detection.

A further source of optimism has emerged from the integration of metabolomics with artificial intelligence and machine learning (ML) approaches. These computational methods have shown potential for identifying complex metabolic patterns that may be difficult to recognize using conventional statistical techniques. Nevertheless, most published models remain at the proof of concept stage and often suffer from limited sample sizes, inadequate external validation, and concerns regarding reproducibility and clinical interpretability [9, 10]. Consequently, the gap between technological innovation and clinical implementation remains substantial.

This review critically examines the evolving landscape of metabolomics in PDAC detection, with particular emphasis on NMR based approaches and their role in distinguishing malignant from benign pancreatic conditions. We provide a balanced evaluation of the biological insights, diagnostic opportunities, and translational challenges associated with metabolomic biomarkers while assessing whether current advances are sufficient to meaningfully improve clinical diagnosis or whether significant methodological and validation barriers must first be overcome before widespread clinical adoption can be achieved.

## 2 Metabolomics in Pancreatic Cancer: Identifying Novel Biomarkers for Early Detection, A Critical Assessment

Metabolomics has emerged as a promising approach for identifying novel biomarkers for the early detection of PDAC, primarily because metabolic alterations frequently precede overt clinical symptoms and radiological manifestations. Nevertheless, despite substantial advances in metabolite discovery, the translation of metabolomic findings into clinically applicable diagnostic tools remains limited by significant methodological and biological challenges. Current metabolomic investigations predominantly rely on three analytical platforms: liquid chromatography‐mass spectrometry (LC‐MS/MS), gas chromatography‐mass spectrometry (GC‐MS), and NMR spectroscopy. LC‐MS/MS offers exceptional sensitivity and broad metabolite coverage for untargeted analyses [11], GC‐MS provides robust quantification of volatile and derivatized metabolites [12], and NMR enables highly reproducible and non‐destructive metabolite profiling [13]. However, this technological diversity has also created substantial challenges for standardization. Differences in sample preparation, analytical workflows, metabolite coverage, and data normalization strategies frequently generate inconsistent biomarker signatures across studies, thereby limiting reproducibility and external validation [14]. Furthermore, variations in cohort composition, disease stage distribution, dietary habits, medication use, and clinical confounders complicate direct comparisons between studies and hinder the establishment of universally applicable diagnostic thresholds [14].

The biological insights generated through metabolomic investigations have substantially advanced our understanding of PDAC‐associated metabolic reprogramming. Alterations in amino acid metabolism, including elevated aspartate and glutamate levels together with disrupted glutathione cycling, indicate increased oxidative stress and dysregulation of the γ‐glutamyl cycle during early tumorigenesis [15]. Likewise, perturbations in central carbon metabolism, characterized by accumulation of tricarboxylic acid (TCA) cycle intermediates such as malate and fumarate accompanied by oxaloacetate depletion, suggest diversion of metabolic flux toward anabolic pathways that support rapid cellular proliferation [16]. These observations highlight the profound metabolic rewiring that occurs during PDAC development and provide a strong biological rationale for biomarker discovery.

Recent studies have further strengthened the diagnostic potential of metabolomics. Untargeted metabolomic profiling of resectable PDAC identified a three‐metabolite signature consisting of 16‐hydroxypalmitic acid, phenylalanine, and norleucine that demonstrated excellent discriminatory performance between patients and controls [17]. Importantly, diagnostic accuracy improved further when the metabolite panel was combined with CA19‐9 [17], suggesting that metabolomic biomarkers may currently provide greater value as complementary rather than replacement diagnostic tools. Emerging evidence also indicates that lipid metabolism abnormalities may have both diagnostic and prognostic significance. Reduced serum high‐density lipoprotein cholesterol (HDL‐C) concentrations have been associated with PDAC and correlated with histological grade, supporting the growing recognition that lipid‐related metabolic alterations contribute to disease progression and may serve as clinically relevant biomarkers [18, 19].

Despite these promising findings, the overall strength of evidence remains uneven. Most candidate biomarkers have been identified in relatively small, single‐center cohorts and frequently lack independent external validation. Moreover, several metabolic alterations reported in PDAC, including disturbances in amino acid, glycolytic, and lipid pathways, are not unique to pancreatic cancer and may also occur in chronic pancreatitis, diabetes mellitus, obesity, and other inflammatory conditions [15, 16]. Consequently, biomarkers demonstrating excellent performance in discovery cohorts often exhibit diminished diagnostic accuracy when evaluated in larger and more heterogeneous populations. These observations highlight the ongoing challenge of distinguishing biologically meaningful disease‐specific signatures from broader systemic metabolic disturbances.

When critically compared with existing diagnostic modalities, metabolomics offers several theoretical advantages while also facing important limitations. Unlike imaging techniques, which primarily detect structural abnormalities, or ctDNA assays that capture genomic alterations, metabolomics provides a dynamic representation of tumor‐associated biochemical activity. However, whether these metabolic signatures consistently provide incremental diagnostic value beyond established multimodal diagnostic strategies remains insufficiently demonstrated. Similarly, comparisons with CA19‐9 require careful interpretation. Although numerous studies have reported superior diagnostic performance for specific metabolite panels, particularly in early‐stage disease, these findings have not yet been consistently reproduced across independent cohorts. In many instances, the greatest diagnostic benefit has been achieved through combining metabolomic signatures with CA19‐9 rather than replacing conventional biomarkers altogether [17].

Overall, metabolomics has substantially expanded our understanding of PDAC‐associated metabolic vulnerabilities and generated a growing repertoire of candidate biomarkers for early detection. Nevertheless, significant barriers remain, including analytical heterogeneity, biological variability, batch effects, inter‐laboratory reproducibility issues, and the lack of prospective multicenter validation studies [14]. Future progress will depend not only on identifying additional biomarkers but also on establishing standardized preprocessing workflows, harmonized analytical protocols, and rigorous external validation frameworks. Only through coordinated multicenter efforts can metabolomics transition from generating promising biomarker candidates to delivering clinically actionable diagnostic tools for early PDAC detection.

## 3 Metabolite Profiling in Blood and Urine as Non‐Invasive Diagnostic Tools

The pursuit of non‐invasive diagnostic strategies for pancreatic cancer has rightly intensified, with blood and urine emerging as primary biofluids for metabolomic analysis. However, a critical examination reveals that both matrices present distinct yet complementary challenges and opportunities that must be carefully weighed for meaningful clinical translation.

Blood‐based metabolomics undoubtedly provides a more stable and systemic metabolic snapshot, as evidenced by the Japan Public Health Center study where elevated plasma branched‐chain amino acids predicted pancreatic cancer risk over a decade before diagnosis [20]. This stability is further corroborated by studies demonstrating blood's superior performance over urine in discriminating inflammatory conditions and detecting ctDNA methylation changes [21, 22]. Yet this systemic perspective comes at the cost of invasiveness and may dilute localized tumor‐specific signatures through homeostatic mechanisms. Moreover, although blood‐based metabolomics is generally considered more amenable to clinical implementation than urine‐based approaches, substantial variability in sample handling, storage conditions, and analytical preprocessing continues to affect reproducibility across laboratories, limiting direct comparison of reported biomarker panels.

Conversely, urine metabolomics offers complete non‐invasiveness and appears particularly adept at capturing specific pathological signatures, achieving remarkable 94% specificity for pancreatic cancer detection through lipid profiling [23]. The elevation of urinary CRP independent of disease stage further underscores urine's capacity to reflect local pathophysiological changes [24]. However, these encouraging findings should be interpreted cautiously, as most urinary biomarker studies have been conducted in relatively small cohorts and rarely include independent external validation. Furthermore, the higher inter‐individual variability driven by environmental factors such as diet, hydration status, medication use, and renal function presents substantial challenges for standardization and reproducibility across diverse populations [21].

The most compelling evidence emerges from integrated approaches. Zong et al. [25] demonstrated that serum metabolites correlate strongly with systemic parameters such as eGFR, whereas urine metabolites better reflect local physiological conditions including albuminuria. This biofluid‐specific metabolic partitioning is further supported by multi‐center analyses of nearly 8000 case‐control pairs revealing conserved yet distinct metabolic disruptions across cancers [26]. The finding that mannose in serum and sebacate in urine independently predict disease progression highlights the complementary nature of these biofluids and suggests that integrated biomarker models may provide greater diagnostic utility than either matrix alone.

Nevertheless, several critical limitations persist. The field lacks standardized protocols for normalizing urinary metabolite concentrations against physiological variables. In addition, differences in sample collection procedures, metabolite extraction methods, analytical platforms, normalization strategies, and batch‐correction workflows contribute substantially to inter‐study variability, complicating efforts to establish universally applicable diagnostic thresholds. Most studies reporting high diagnostic accuracy for urinary biomarkers remain single‐center investigations without external verification. Furthermore, the biological mechanisms underlying the compartmentalization of metabolic signatures between blood and urine remain poorly understood, limiting rational biomarker selection and mechanistic interpretation.

When evaluated against clinical needs, the current evidence suggests that blood‐based approaches may be more immediately translatable for population screening because of superior standardization and existing clinical infrastructure, whereas urine‐based strategies offer potential advantages for longitudinal monitoring and personalized applications once standardization challenges are addressed. Nevertheless, whether metabolomic profiling provides sufficient incremental diagnostic value beyond established modalities such as CA19‐9, imaging‐based surveillance, and emerging ctDNA assays remains uncertain and requires validation in large prospective studies. The promising diagnostic accuracy achieved by combining blood‐ and urine‐derived metabolic signatures further underscores the limitations of relying on either biofluid alone [25].

In conclusion, while both blood and urine metabolomics offer valuable insights into PDAC‐associated metabolic alterations, their true clinical potential will likely be realized through integrated analytical frameworks that leverage the complementary strengths of both biofluids. Future research should prioritize elucidating the biological basis of biofluid‐specific metabolic signatures, improving inter‐laboratory reproducibility, and establishing standardized protocols capable of supporting reliable multi‐matrix integration for early pancreatic cancer detection.

## 4 Signaling Metabolites in Pancreatic Cancer: From Bench to Bedside, Assessing the Translational Gap

**Figure 2:**
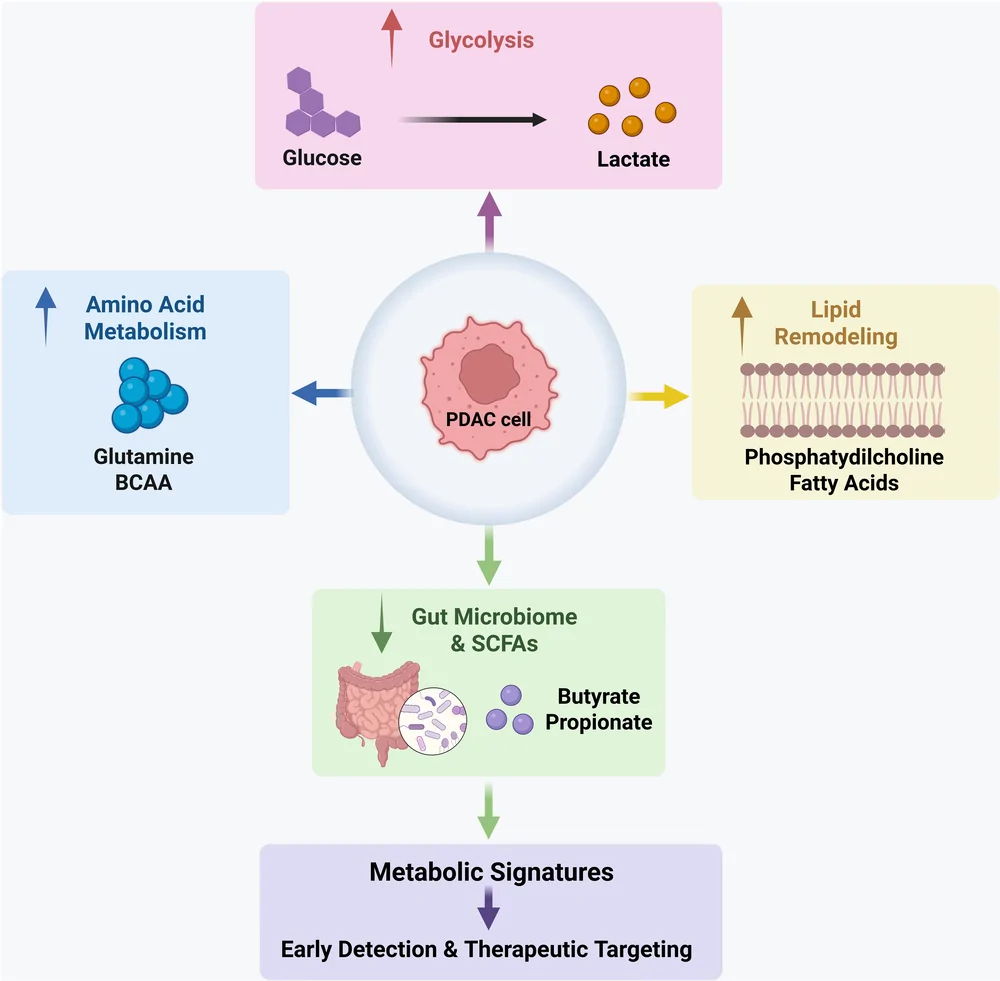
Major metabolic pathways altered in PDAC. PDAC is characterized by enhanced glycolysis, increased amino acid utilization, and extensive lipid remodeling, accompanied by reduced levels of gut microbiome‐derived short‐chain fatty acids (SCFAs), including butyrate and propionate. Together, these interconnected metabolic alterations contribute to tumor progression and generate distinct metabolic signatures that may serve as potential biomarkers for early detection and therapeutic targeting.

PDAC undergoes extensive metabolic reprogramming involving multiple interconnected pathways, including enhanced glycolysis, altered amino acid metabolism, lipid remodeling, and dysregulation of gut microbiome‐derived metabolites (Figure 2). These metabolic alterations not only support tumor growth and progression but also provide a rich source of potential diagnostic biomarkers and therapeutic targets. However, translating these mechanistic insights into clinically actionable strategies remains challenging. The well‐characterized Warburg effect and glutamine addiction indeed define PDAC metabolism, with lactate emerging as a master signaling metabolite rather than a mere waste product. The LOXL2‐HIF1α axis drives substantial lactate production [27], creating an acidic microenvironment (pH 6.0–6.5) that actively promotes immunosuppression and metastasis [28, 29]. Simultaneously, glutamine addiction redirects nitrogen metabolism toward biomass production [30, 31]. Beyond their functional role in tumor progression, these metabolic alterations have attracted attention as potential biomarkers of aggressive disease behavior and therapeutic response. However, the extent to which signaling metabolites identified in experimental systems retain diagnostic or prognostic utility in heterogeneous patient populations remains insufficiently established. While these mechanisms are robustly supported by preclinical evidence, their clinical relevance requires careful scrutiny given the complexity of in vivo metabolic interactions.

The therapeutic strategies derived from these metabolic insights, including targeting lactate dynamics through LDH and MCT inhibition, exploiting glutamine addiction with glutaminase inhibitors, and modulating sirtuin activity at the metabolism–epigenetics interface [32, 33], demonstrate compelling preclinical efficacy. However, this promise must be tempered by the sobering reality of translational barriers. The fundamental challenge lies in the metabolic plasticity of PDAC cells, which rapidly develop resistance through pathway redundancy and adaptive metabolic rewiring. This plasticity is further complicated by the dense stromal architecture of PDAC that creates physical and chemical barriers to drug delivery.

Critical limitations persistently hinder bedside translation. Therapeutic window constraints remain a major obstacle because many metabolic targets are also essential for normal cellular function, resulting in narrow therapeutic indices that restrict effective dosing. Compounding this challenge are biomarker deficiencies; the absence of reliable biomarkers for patient stratification means that clinical trials often include substantial proportions of non‐responders, thereby diluting efficacy signals. Notably, many candidate metabolic biomarkers proposed for therapeutic stratification have yet to undergo prospective validation, highlighting a persistent disconnect between biomarker discovery and clinical implementation. Additionally, significant metabolic heterogeneity within tumors enables rapid adaptive resistance through compensatory pathways that bypass single‐metabolite or single‐enzyme inhibition.

The ongoing early‐phase clinical trials of LDH and MCT inhibitors confront these challenges directly, with their modest results to date underscoring the substantial gap between mechanistic understanding and clinical application. The field has arguably underestimated the robust compensatory networks that allow PDAC cells to circumvent single‐pathway inhibition, suggesting that rational combination approaches may be essential for meaningful clinical impact. Furthermore, the lack of standardized metabolomic assays, clinically validated cutoff values, and harmonized analytical workflows limits the integration of signaling metabolites into routine therapeutic decision‐making and clinical trial design.

When critically evaluated, the bench‐to‐bedside narrative requires significant qualification. While metabolomics has unequivocally revealed lactate and glutamine as active signaling drivers, translating this knowledge into effective therapies demands solutions to fundamental biological and pharmacological challenges. The field must evolve beyond target identification to address delivery limitations, resistance mechanisms, and patient selection strategies. Future success will likely depend on developing multimodal approaches that simultaneously target multiple metabolic vulnerabilities while accounting for tumor heterogeneity and microenvironmental influences.

In conclusion, while signaling metabolites represent biologically validated therapeutic targets in PDAC, their successful clinical translation necessitates innovative strategies to overcome metabolic plasticity, enhance drug delivery, and identify predictive biomarkers. The therapeutic potential remains substantial, but its realization hinges on addressing critical challenges related to validation, standardization, and patient selection through integrated multidisciplinary efforts that bridge basic science and clinical medicine.

## 5 Fatty Acids and Lipid Metabolites as Diagnostic Markers in PDAC: Promise and Pitfalls

Distinct lipidomic alterations have emerged as a hallmark of PDAC, positioning lipid metabolites as promising diagnostic biomarkers while also revealing their active roles in tumor progression. However, the translation of these findings into clinically applicable tools faces substantial challenges that warrant critical examination. Phospholipid metabolism undergoes dramatic shifts characterized by elevated phosphatidylcholine levels driven by choline kinase alpha overexpression [34], while sphingolipid alterations, particularly an imbalanced ceramide‐to‐sphingomyelin ratio, contribute to chemoresistance through activation of pro‐survival signaling pathways [35]. Emerging clinical evidence further suggests that systemic lipid abnormalities may carry both diagnostic and prognostic significance. Reduced serum HDL‐C concentrations have been associated with PDAC and correlate with unfavorable histological grade, highlighting the broader relevance of lipid dysregulation beyond tumor metabolism [18, 19]. The obesity epidemic further complicates this landscape by exacerbating metabolic dysfunction through adipokine dysregulation and chronic inflammation, both of which promote lipid accumulation and metabolic adaptation in cancer cells [36, 37].

The diagnostic potential of lipid metabolites is compelling but requires careful interpretation. Multi‐omic studies have identified extensive lipidomic alterations, with comprehensive blood‐based analyses detecting 873 significantly altered lipids associated with dysregulated glycolipid metabolism pathways [38]. While such findings illustrate the richness of the lipidome as a source of candidate biomarkers, the clinical utility of large biomarker panels remains uncertain. ML‐assisted approaches have generated promising results, including a 38‐biomarker signature for early PDAC detection and a three‐metabolite panel consisting of proline, creatine, and palmitic acid that achieved AUC values ranging from 0.83 to 0.865 [39]. Furthermore, combining lipid‐derived biomarkers with CA19‐9 substantially improved diagnostic performance (AUC: 0.909–0.949) [39], supporting the concept that lipidomics may complement rather than replace established diagnostic markers. Although a systematic meta‐analysis confirmed the overall discriminatory value of metabolite‐based biomarkers (AUC = 0.86) [40], many of the reported high‐performing panels have been evaluated primarily in discovery cohorts and frequently lack independent external validation. Consequently, the impressive diagnostic metrics reported in individual studies may overestimate real‐world performance when applied to larger and more heterogeneous patient populations.

The gut microbiome introduces an additional layer of complexity to lipid metabolism in PDAC. Distinct microbial shifts in patients with advanced disease, including reductions in *Alistipes* and *Anaerostipes* and increases in *Cloacibacterium* and *Mucispirillum*, have been associated with alterations in fatty acids and carnitine derivatives [41]. Specific lipid metabolites, including oleic acid, linoleic acid, and palmitic acid, have demonstrated strong discriminatory performance (AUC > 0.9) and have been linked to oncogenic signaling pathways such as NF‐κB and mTOR [41]. However, the causal relationship between microbiome‐associated lipid alterations and PDAC progression remains incompletely understood. Most available evidence is observational, making it difficult to determine whether these metabolic changes actively contribute to tumor progression or simply reflect disease‐associated physiological disturbances. Therefore, while microbiota‐metabolite interactions offer intriguing opportunities for disease stratification, their clinical applicability should be interpreted cautiously until mechanistic and longitudinal validation studies become available.

Beyond diagnostics, lipid alterations provide important insights into potential therapeutic vulnerabilities. Sphingosine kinase 1 has emerged as both a candidate biomarker and a potential therapeutic target [40], while integrated multi‐omics analyses have identified 517 differentially expressed genes linked to lipid metabolic pathways [40]. Nevertheless, the progression from molecular characterization to effective targeted therapies has been slow. This discrepancy underscores a broader challenge within PDAC research: the ability to identify biologically relevant metabolic alterations has outpaced the development of clinically actionable interventions. As a result, the translational impact of many lipid‐associated discoveries remains uncertain.

Substantial challenges persist in standardizing lipidomic workflows, including sample collection procedures, lipid extraction methods, analytical platforms, data preprocessing strategies, batch‐effect correction, and inter‐laboratory harmonization. Biological variability introduced by dietary habits, obesity, medication use, and metabolic comorbidities further complicates interpretation and reproducibility. Moreover, the limited overlap among lipid biomarkers identified in independent cohorts raises concerns regarding the robustness and generalizability of proposed diagnostic signatures. While combining lipid metabolites with conventional biomarkers and integrating lipidomics into broader multi‐omic frameworks holds considerable promise, successful clinical implementation will require rigorous standardization, prospective multicenter validation, demonstration of cost‐effectiveness, and seamless integration into existing diagnostic workflows.

In conclusion, lipid metabolites represent valuable but imperfect tools within the evolving diagnostic landscape of PDAC. Although substantial progress has been made in characterizing lipid metabolic alterations and identifying promising biomarker candidates, significant barriers to clinical translation remain. Future efforts should prioritize standardized analytical methodologies, external validation across diverse populations, and careful evaluation of the incremental value of lipidomics relative to existing diagnostic modalities. Ultimately, the field must move beyond cataloging lipid alterations toward demonstrating tangible improvements in early detection, risk stratification, and patient outcomes.

## 6 Alterations in Glycolysis‐Related Metabolites in Pancreatic Cancer: Diagnostic Promise versus Therapeutic Reality

PDAC exhibits profound metabolic reprogramming characterized by markedly enhanced glycolytic activity, representing one of the most consistently validated metabolic hallmarks in cancer biology. The Warburg effect drives glucose uptake rates up to 30‐fold higher than those observed in normal pancreatic tissue [42], primarily orchestrated by oncogenic KRAS mutations through MAPK signaling pathways [43, 44]. This molecular framework manifests as substantial metabolic alterations, including 3–5‐fold GLUT1 overexpression [45, 46], approximately eightfold increases in HK2 activity [42], and fourfold elevations in LDHA expression [47, 48]. Collectively, these changes generate a characteristic glycolytic phenotype that has shown promise in distinguishing PDAC from benign pancreatic disorders, including chronic pancreatitis.

The diagnostic implications of glycolytic dysregulation are substantial yet require careful interpretation. Serum lactate levels have been reported to increase approximately 2.8‐fold in PDAC compared with benign conditions [49, 50], whereas pyruvate concentrations decrease by nearly 40% [50]. NMR‐based metabolomic profiling has exploited this lactate‐to‐pyruvate imbalance, achieving impressive diagnostic performance (AUC = 0.956) through panels incorporating elevated glucose and reduced citrate levels [50]. Nevertheless, many high‐performing metabolite signatures have been derived from relatively small cohorts and remain insufficiently validated in independent multicenter populations. Consequently, their reported diagnostic performance should be interpreted cautiously until reproducibility across diverse clinical settings has been demonstrated. Furthermore, the underlying metabolic signatures reflect dynamic tumor‐associated processes in which cancer cells convert a substantial proportion of absorbed glucose into lactate [51], creating an acidic microenvironment (pH ~6.5) that promotes immune evasion and metastatic progression [49, 52]. While biologically informative, these processes may also introduce significant inter‐patient variability that complicates clinical implementation.

Emerging imaging modalities provide additional insight into glycolytic dysregulation. Hyperpolarized carbon‐13 (^13^C) MRI enables real‐time assessment of pyruvate‐to‐lactate conversion [53], while 18F‐FDG PET remains the most widely used metabolic imaging technique, despite limited specificity in distinguishing PDAC from inflammatory pancreatic conditions [54, 55, 56].

The therapeutic exploitation of glycolytic dependencies highlights a persistent gap between experimental success and clinical implementation. Glycolytic inhibitors such as 2‐deoxyglucose demonstrate substantial reductions in PDAC cell viability and enhanced responses to gemcitabine in preclinical models [57]. Likewise, LDHA‐targeted approaches have shown encouraging results in KRAS‐driven experimental systems [44, 49]. However, these findings have not translated consistently into meaningful clinical benefit. A major challenge is the considerable metabolic heterogeneity observed among PDAC tumors, with approximately 30% of cases exhibiting alternative metabolic adaptations that reduce dependence on canonical glycolytic pathways [58]. This variability not only limits the effectiveness of single‐pathway interventions but also underscores the need for metabolomic stratification approaches capable of identifying patients most likely to benefit from glycolysis‐targeted therapies.

Several critical questions remain unresolved. These include the extent of metabolic heterogeneity among PDAC subtypes [59], the precise immunomodulatory consequences of lactate accumulation [49, 60], and the optimal integration of metabolic imaging into disease monitoring and therapeutic assessment [61, 62]. Although glycolysis‐related metabolites remain attractive candidates for both diagnosis and therapeutic targeting, their successful clinical translation will require standardized analytical methodologies, harmonized preprocessing workflows, and rigorous multicenter validation. The strong performance of NMR‐based metabolite panels suggests potential for complementing existing imaging modalities and CA19‐9 measurements. Notably, recent metabolomic investigations have demonstrated that combining metabolite signatures with CA19‐9 improves diagnostic performance compared with either approach alone, supporting a multimodal rather than replacement‐based diagnostic strategy [17].

Overall, glycolytic metabolites remain among the most extensively validated metabolic alterations in PDAC biology. However, future progress will likely depend less on identifying additional glycolytic abnormalities and more on overcoming the technical, biological, and translational barriers that currently limit clinical implementation. Achieving this goal will require improved standardization, external validation, patient stratification strategies, and integration with existing diagnostic and therapeutic frameworks to fully realize the clinical potential of glycolysis‐based biomarkers.

## 7 Gut Microbiome‐Derived Metabolites in Pancreatic Cancer: Therapeutic Promise versus Measurement Challenges

The gut microbiome‐derived short‐chain fatty acids (SCFAs), including acetate, propionate, and butyrate, represent a compelling yet complex dimension of PDAC pathophysiology. While these metabolites, produced through bacterial fermentation of dietary fiber by commensals such as Lachnospiraceae and Ruminococcaceae [63], undoubtedly play important roles in maintaining intestinal barrier integrity and regulating immune responses [64, 65], their precise role in PDAC progression demands more nuanced interpretation. The documented alterations observed in PDAC patients, including a 60% reduction in fecal butyrate and a 45% decrease in propionate compared with healthy controls [66, 67], correlate with a 70% decline in butyrate‐producing bacteria such as *Roseburia* and *Faecalibacterium* [68]. However, the causal relationship between these microbial shifts and tumor progression remains inadequately established, leaving open the possibility that these changes may represent consequences rather than drivers of malignancy. Furthermore, reverse causation remains a significant concern, as tumor‐associated metabolic alterations, dietary changes, pancreatic exocrine insufficiency, and treatment‐related effects may themselves reshape the gut microbiome, thereby complicating causal inference.

The purported anti‐tumor mechanisms of SCFAs, while mechanistically intriguing, require critical appraisal within the context of PDAC's unique microenvironment. Butyrate demonstrates growth‐inhibitory effects in cultured PDAC cells and promotes cellular differentiation [8], while enhancing ferroptosis susceptibility through oxidative stress and lipid peroxidation [69]. The reported 30% increase in ferroptosis susceptibility following *Clostridium butyricum* supplementation [69] and the 40% enhancement of gemcitabine efficacy with butyrate co‐treatment [70] suggest potentially valuable therapeutic avenues. Similarly, propionate‐mediated reductions in tumor growth observed in murine models [71] indicate broader anti‐tumor effects of SCFAs. Nevertheless, these promising findings derive predominantly from experimental systems, and substantial biological barriers remain to achieving therapeutically effective intratumoral SCFA concentrations in human patients.

The immunomodulatory effects of SCFAs present both opportunity and complexity. Butyrate has been shown to increase regulatory T‐cell differentiation threefold [72] and reduce macrophage IL‐6 production by approximately 60% [73], mechanisms that may influence inflammatory signaling within the tumor microenvironment. Furthermore, the inverse correlation observed between butyrate‐producing bacteria and the neutrophil‐to‐lymphocyte ratio (*r* = −0.62) [74] suggests potential clinical relevance. However, the dual nature of these immunomodulatory effects complicates therapeutic translation, as SCFAs may simultaneously suppress pro‐tumor inflammation while also dampening anti‐tumor immune responses. Consequently, the net impact of SCFA modulation on PDAC progression remains insufficiently understood.

From a diagnostic perspective, SCFA profiling demonstrates intriguing potential but faces formidable technical obstacles. Fecal butyrate concentrations have been reported to distinguish PDAC from autoimmune pancreatitis with approximately 82% accuracy [66, 67], while serum SCFA‐based models have achieved AUC values exceeding 0.80 [75, 76]. Despite these encouraging results, methodological challenges remain substantial. GC‐MS‐based quantification methods exhibit variable recovery rates ranging from 90% to 117% [75], while physiological factors such as the limited fecal excretion of SCFAs and poor intra‐individual consistency in circulating SCFA concentrations (ICC < 0.4) [77, 78] complicate clinical interpretation. Additional challenges arise from differences in sample collection procedures, storage conditions, metabolite extraction methods, analytical platforms, and data preprocessing pipelines, all of which contribute to substantial inter‐study variability and hinder reproducibility. Consequently, these technical limitations continue to impede reliable standardization across research centers and clinical settings.

The translational pathway for SCFA‐based interventions encounters multiple critical barriers. Determining optimal supplementation strategies requires addressing fundamental questions regarding delivery methods, dosing regimens, treatment duration, and patient stratification. Furthermore, understanding how dietary interventions influence microbial SCFA production in PDAC patients will require carefully controlled clinical trials capable of accounting for extensive inter‐individual variability in microbiome composition and metabolic response. Moreover, the clinical deployment of microbiome‐based metabolomic testing will require cost‐effective analytical workflows, specialized laboratory infrastructure, and clearly defined regulatory pathways before routine implementation can be considered.

In conclusion, while SCFAs represent biologically plausible diagnostic biomarkers and potential therapeutic targets, their clinical utility remains constrained by methodological challenges and an incomplete understanding of their role in PDAC pathogenesis. Future research should prioritize overcoming technical measurement barriers, improving inter‐laboratory reproducibility, validating findings in prospective multicenter cohorts, and developing targeted delivery approaches capable of achieving therapeutically relevant SCFA concentrations within the tumor microenvironment. Ultimately, the considerable promise of the gut microbiome–SCFA axis must be balanced against the substantial scientific and translational hurdles that currently limit its clinical application in pancreatic cancer management. Collectively, these findings highlight the interconnected nature of glycolytic, amino acid, lipid, and microbiome‐associated metabolic alterations in PDAC, as summarized in Figure 2.

## 8 MS in Metabolite Discovery: Capabilities, Limitations, and Clinical Translation Challenges

MS has undoubtedly revolutionized metabolomics, enabling comprehensive analysis of metabolites with unprecedented sensitivity and specificity [79, 80]. However, a critical examination reveals that the two dominant MS platforms, GC‐MS and LC‐MS, present important trade‐offs that influence their utility in PDAC biomarker discovery. While these approaches are often portrayed as complementary technologies [81, 82], their fundamental analytical differences frequently generate non‐overlapping datasets, complicating cross‐study comparisons, biomarker validation, and the establishment of universally applicable diagnostic signatures.

GC‐MS represents a mature analytical platform characterized by excellent chromatographic reproducibility and access to well‐established spectral libraries [83]. These features make it particularly valuable for investigating volatile compounds and metabolites involved in primary metabolic pathways. Nevertheless, the requirement for chemical derivatization of many polar metabolites [84] introduces additional sources of technical variability, while limitations in analyzing thermally labile compounds and relatively long analytical run times restrict its suitability for high‐throughput clinical applications [83, 84]. Consequently, despite its robust quantitative performance [85], GC‐MS provides only partial coverage of the metabolome and may overlook biologically relevant metabolic alterations associated with PDAC progression.

LC‐MS has substantially expanded metabolomic capabilities by enabling the analysis of non‐volatile and thermally unstable metabolites [86]. Advances such as ultra‐performance liquid chromatography coupled with high‐resolution mass spectrometry (UPLC‐HRMS) have improved both analytical throughput and metabolite coverage [87, 88]. The ability to perform rapid analyses within a few minutes makes LC‐MS particularly attractive for translational research and potential clinical implementation. However, matrix effects, ion suppression, variability in metabolite ionization efficiency, and the absence of universally accepted spectral reference libraries remain major obstacles to biomarker verification and inter‐laboratory reproducibility [85, 87]. These challenges contribute to the persistent difficulty of reproducing proposed biomarker panels across independent cohorts.

Recent technological developments, including high‐resolution accurate‐mass instruments with sub‐ppm mass accuracy [89, 90], ion mobility separation technologies [88], and data‐independent acquisition approaches such as SWATH‐MS [91, 92], have further enhanced analytical performance. However, these advances have also increased the complexity of metabolomic data interpretation. ML‐assisted annotation and metabolite identification workflows remain constrained by incomplete reference databases, feature redundancy, limited external validation, and the risk of model overfitting when applied to high‐dimensional metabolomic datasets [93]. Consequently, improvements in analytical sensitivity have not necessarily translated into equivalent gains in biological interpretation or clinical utility.

In pancreatic cancer research, MS‐based metabolomics has identified significant alterations in bile acid, fatty acid, and amino acid metabolism through LC‐MS analyses [91, 92], as well as perturbations in glycolysis and TCA cycle intermediates using GC‐MS approaches [84]. These discoveries have substantially improved understanding of PDAC‐associated metabolic reprogramming. Nevertheless, the translation of such findings into clinically useful biomarkers has been hindered by persistent challenges in sample preparation standardization, protocol variability across laboratories, metabolite annotation discrepancies, and the inherent mismatch between biological complexity and analytical capabilities [94, 95]. As a result, relatively few candidate biomarkers have progressed beyond discovery‐stage investigations.

Beyond analytical performance, the routine clinical adoption of MS‐based metabolomics will depend on practical considerations including instrument cost, maintenance requirements, technical expertise, turnaround time, laboratory infrastructure, and regulatory approval pathways. These factors remain significant barriers to implementation, particularly in resource‐limited healthcare settings. Therefore, demonstrating analytical excellence alone is insufficient; successful clinical translation will require evidence that MS‐based metabolomic testing can be implemented in a cost‐effective and operationally feasible manner.

Importantly, MS‐based metabolomics should be viewed as complementary to, rather than a replacement for, established diagnostic approaches such as CA19‐9, imaging modalities, and emerging liquid biopsy technologies. The greatest clinical benefit may ultimately arise from integrated diagnostic frameworks that combine metabolic, molecular, and radiologic information, thereby leveraging the strengths of multiple modalities while minimizing their individual limitations.

The future development of more comprehensive spectral libraries and improved data‐sharing infrastructures represents an important step forward, but these advances alone are unlikely to resolve the major translational challenges facing the field. Meaningful progress will require recognition that technological sophistication cannot fully overcome the biological heterogeneity and metabolic complexity of PDAC [96]. Consequently, continued instrument evolution and integration with other omics technologies must be accompanied by rigorous clinical validation frameworks, harmonized pre‐analytical procedures, and multicenter reproducibility studies [88, 97].

In conclusion, while MS technologies provide exceptionally powerful tools for metabolite discovery in PDAC, their clinical translation remains constrained by technical variability, analytical limitations, standardization challenges, and insufficient validation in clinically relevant populations. Future efforts should prioritize method harmonization, independent verification of proposed biomarkers, demonstration of clinical utility, and integration into practical diagnostic workflows. Ultimately, the field requires not only continued technological innovation but also a stronger emphasis on overcoming the biological, logistical, and regulatory barriers that currently limit the routine clinical application of MS‐based metabolomics in pancreatic cancer diagnosis and monitoring.

## 9 Integrating Metabolomics and ML for Early Detection: Navigating the Hype and Reality

The integration of metabolomics and ML represents a compelling paradigm for early PDAC detection, yet a critical assessment reveals substantial gaps between computational promise and clinical deliverability. While the theoretical synergy is undeniable, metabolomics provides rich biochemical information whereas ML offers sophisticated pattern recognition capabilities [98, 99]. However, practical implementation faces fundamental methodological and translational challenges that are often understated in the literature.

The reported successes, although encouraging, require careful scrutiny. Wang et al.'s LC‐MS lipidomic panel achieved an accuracy of 86.74% with an AUC of 0.9351 [100], while a 38 biomarker multiomics signature demonstrated similarly promising performance [32]. These findings illustrate the potential of data driven biomarker discovery, yet validation in large independent multicenter cohorts remains limited. Among available algorithms, tree‐based methods such as Random Forest and XGBoost appear particularly effective in handling nonlinear relationships and complex metabolomic datasets [101, 102], whereas Support Vector Machines continue to perform well in settings characterized by limited sample sizes and large feature spaces. In contrast, enthusiasm surrounding deep learning approaches should be tempered by the reality that most metabolomic studies lack sufficiently large datasets to support robust neural network training, raising concerns regarding generalizability and practical utility in current PDAC research settings [103].

A central challenge remains the well‐recognized curse of dimensionality, whereby thousands of metabolic features are analyzed in cohorts consisting of relatively few patients [104]. This imbalance creates persistent risks of overfitting, spurious associations, and inflated estimates of predictive performance. Robust model development therefore requires clear separation of training and test datasets, rigorous cross validation strategies, and independent external validation cohorts. Failure to implement these safeguards may result in data leakage and overly optimistic performance estimates that are unlikely to generalize to real world clinical populations. Although feature selection methods such as LASSO regression [100, 105] and dimensionality reduction approaches can mitigate some of these issues, they may also introduce bias and potentially eliminate biologically meaningful signals in pursuit of statistical stability [106]. Furthermore, class imbalance remains a recurrent challenge in PDAC studies, particularly when early‐stage tumors constitute only a small proportion of available datasets. Techniques such as stratified sampling, synthetic minority oversampling, and cost sensitive learning may improve model stability, although inappropriate implementation can itself introduce additional sources of bias. Equally important is the challenge of data preprocessing and normalization, where technical variation arising from sample handling, batch effects, or analytical platforms may be inadvertently interpreted by algorithms as biologically meaningful patterns [107].

Emerging solutions offer opportunities for improvement but also introduce new complexities. Ensemble learning approaches often enhance model robustness at the expense of interpretability, while transfer learning strategies face significant challenges related to domain adaptation across different populations and disease contexts [102]. Most concerning is the ongoing tension between predictive performance and clinical interpretability. Although explainable AI techniques, including SHAP values and related approaches, have improved transparency [108, 109, 110], many of the highest performing models remain difficult to interpret biologically. In addition, calibration is frequently underreported in metabolomics‐based ML studies. Consequently, models demonstrating excellent discrimination may still generate poorly calibrated risk estimates, limiting their value in clinical decision making and patient management.

The clinical applications of metabolomics guided ML remain promising but continue to suffer from significant validation deficits. High performing models, including miRNA‐based classifiers with reported accuracies approaching 93% and metabolomic signatures distinguishing PDAC from chronic pancreatitis with approximately 82% accuracy, illustrate the potential of these approaches. However, most published models have undergone only internal validation, and relatively few have been evaluated in independent prospective cohorts. Consequently, the apparent superiority of many ML based approaches remains uncertain when assessed under real world clinical conditions. Recent untargeted metabolomic investigations have further demonstrated the potential of integrated metabolic signatures for PDAC detection, reinforcing the value of computational approaches while simultaneously highlighting the need for rigorous validation before clinical deployment [17]. Moreover, claims that ML assisted metabolomic models outperform conventional biomarkers such as CA19‐9 require careful qualification, as many studies compare optimized computational models against CA19‐9 alone rather than against realistic diagnostic workflows that incorporate imaging findings, clinical assessment, and laboratory data simultaneously [38, 111, 112].

Future directions should prioritize practical clinical translation rather than incremental methodological refinement alone. Expanding cohort diversity remains essential but will not fully address the underlying biological heterogeneity of PDAC. Integrating metabolomics with genomic, transcriptomic, proteomic, and radiologic data may improve diagnostic performance, yet such approaches substantially increase analytical complexity and introduce additional challenges related to data harmonization and interpretation. Most importantly, standardized protocols for data acquisition, preprocessing, feature selection, and model validation must be established before large scale prospective clinical studies can generate reliable and reproducible evidence. Equally important is the development of transparent reporting standards that provide detailed descriptions of preprocessing pipelines, feature selection procedures, validation frameworks, and performance metrics to facilitate reproducibility and comparison across studies.

In conclusion, the integration of metabolomics and ML holds genuine promise for transforming early PDAC detection, but substantial methodological and validation hurdles remain. Future research should prioritize rigorous external validation, mitigation of overfitting, management of class imbalance, clinical interpretability, and demonstration of real‐world utility rather than focusing solely on marginal improvements in predictive performance. Ultimately, the success of metabolomics guided ML will be measured not by optimized AUC values in retrospective cohorts, but by its ability to improve clinical decision making, enable earlier diagnosis, and enhance patient outcomes in routine clinical practice.

## 10 NMR Spectroscopy for Discriminating PDAC vs. CP: Analytical Promise versus Clinical Practicality

NMR spectroscopy has gained considerable attention as a valuable analytical platform for differentiating PDAC from chronic pancreatitis [113]. Its ability to directly identify and quantify a broad spectrum of metabolites, including sugars, ketone bodies, amino acids, and organic acids, provides important advantages for metabolic profiling [114, 115]. Nevertheless, the transition from analytical capability to routine clinical implementation remains challenging, and the practical limitations of NMR are often underemphasized relative to its technical strengths.

The development of hybrid NMR and MS platforms represents an important technological advance, combining the quantitative reproducibility of NMR with the superior sensitivity of MS [116]. Although these integrated approaches offer enhanced metabolome coverage, their complexity, operational requirements, and cost currently restrict their use primarily to research environments. Likewise, high resolution magic angle spinning NMR enables rapid tissue characterization within approximately 20 min while preserving sample integrity [117, 118]. However, these advantages must be balanced against the substantial capital investment, specialized infrastructure, and highly trained personnel required for operation.

Several NMR based studies have identified metabolite patterns capable of distinguishing PDAC from chronic pancreatitis. Elevated concentrations of branched chain amino acids, lactate, and 3‐hydroxybutyrate have been consistently reported in PDAC cohorts [119], reflecting broader metabolic reprogramming associated with tumor progression [120]. While these findings provide valuable biological insight, their translation into clinically robust diagnostic algorithms remains limited by biological variability among patients and the relatively modest sensitivity of NMR, which generally operates in the micromolar detection range. In contrast, MS can often detect metabolites at nanomolar or picomolar concentrations, enabling identification of lower abundance biomarkers that may be missed by NMR based approaches [118].

Importantly, NMR offers several practical advantages that continue to support its relevance in clinical metabolomics. The technique is highly reproducible, non‐destructive, and requires relatively limited sample preparation, thereby reducing certain sources of analytical variability [121]. However, these benefits must be weighed against practical implementation challenges, including large instrument footprints, ongoing maintenance costs, specialized operator training, and limited availability outside major academic or tertiary care centers. Consequently, the barriers to widespread adoption extend beyond analytical performance and encompass broader considerations related to healthcare infrastructure and resource allocation.

Emerging tiered diagnostic strategies that combine MS for broad metabolite screening with NMR for confirmatory analysis represent a potentially pragmatic approach to clinical translation [122]. Such workflows may capitalize on the complementary strengths of both technologies while mitigating their individual weaknesses. Nevertheless, reported improvements in turnaround time should be interpreted cautiously and evaluated within the context of the entire diagnostic pathway rather than isolated analytical performance metrics. Furthermore, the cost effectiveness of implementing dual platform diagnostic systems remains uncertain and requires formal evaluation in prospective clinical studies.

From a clinical perspective, NMR should be considered complementary to existing diagnostic modalities rather than a replacement for them. Its greatest potential may lie in integration with established biomarkers such as CA19‐9, imaging techniques, and other metabolomic platforms, where combined approaches may improve diagnostic confidence while reducing the limitations associated with any single method.

In conclusion, NMR spectroscopy provides valuable metabolic information that may aid in distinguishing PDAC from chronic pancreatitis, but its routine clinical implementation remains constrained by limitations related to cost, infrastructure, accessibility, and analytical sensitivity. Future efforts should focus on improving affordability, simplifying operational requirements, enhancing accessibility, and validating clinically actionable metabolite signatures in large multicenter cohorts. Ultimately, the success of NMR based diagnostics will depend not only on analytical performance but also on their ability to deliver practical, cost effective, and scalable solutions within real world healthcare systems.

## 11 Can Specific Metabolites Replace CA19‐9 in Diagnosis? A Critical Pathway Assessment

The search for metabolite‐based biomarkers capable of replacing or supplementing CA19‐9 remains one of the most active areas of PDAC diagnostics. Although CA19‐9 suffers from well recognized limitations, including sensitivity ranging from approximately 60% to 80%, false positive elevations in inflammatory pancreatic diseases, and lack of utility in Lewis antigen negative individuals [123], identifying clinically viable alternatives has proven considerably more difficult than initially anticipated. The absence of any FDA approved metabolomics based diagnostic test for pancreatic cancer highlights the substantial gap between promising research findings and successful clinical translation [124].

Several metabolite panels have demonstrated encouraging diagnostic performance. A multicenter study reported that a 9‐metabolite panel combined with CA19‐9 achieved an AUC of 0.96, while simultaneously reducing both false negative and false positive rates [125]. Similarly, a two‐metabolite signature consisting of isoleucine and adrenic acid achieved AUC values ranging from 0.90 to 0.93 for early stage PDAC detection [126], exceeding the performance commonly reported for CA19‐9 alone. Despite these promising results, it is important to recognize that most studies remain within the discovery or early validation stages. To date, no metabolite panel has successfully progressed through large prospective Phase III validation studies or obtained regulatory approval, raising legitimate concerns regarding reproducibility, generalizability, and real‐world clinical performance [127].

Importantly, growing evidence suggests that metabolomics may provide greater value as a complementary diagnostic modality rather than a direct replacement for existing biomarkers. Recent untargeted metabolomic analyses have demonstrated improved diagnostic performance when metabolite signatures are integrated with established clinical markers, supporting multimodal diagnostic strategies rather than single biomarker approaches [17]. Likewise, studies investigating serum lipid metabolism have identified associations between altered high density lipoprotein cholesterol levels and PDAC characteristics, illustrating the biological relevance of metabolic biomarkers while also highlighting the limitations of relying on individual metabolites as standalone diagnostic tools [18, 19].

Alternative biofluid based approaches, including urinary biomarkers such as LYVE1, REG1A, and TFF1, as well as salivary transcriptomic signatures, further expand the diagnostic landscape [128]. While these approaches offer attractive non‐invasive sampling opportunities, they face many of the same challenges encountered by blood based metabolomic biomarkers, including standardization difficulties, interlaboratory variability, and regulatory requirements. Consequently, the growing number of candidate biomarkers has been accompanied by a parallel increase in validation complexity.

ML and artificial intelligence have introduced additional opportunities for identifying complex metabolic signatures that may be undetectable through conventional statistical approaches. However, these technologies also create new challenges related to model interpretability, external validation, regulatory oversight, and clinical trustworthiness [124]. Proposed diagnostic frameworks that combine MS screening, NMR confirmation, and AI assisted interpretation are conceptually attractive, but their practical implementation remains uncertain without robust prospective validation and demonstration of cost effectiveness across diverse healthcare systems [129].

A balanced comparison with existing diagnostic modalities is essential. Although metabolomic biomarkers frequently outperform CA19‐9 in retrospective research cohorts, clinical diagnosis of PDAC rarely relies on CA19‐9 alone. Instead, diagnostic decision making typically incorporates imaging modalities, histopathological evaluation, and increasingly, liquid biopsy approaches such as ctDNA analysis. Consequently, comparisons between metabolomics and individual diagnostic modalities may overestimate the practical clinical advantage of metabolite‐based tests. The greatest benefit will likely arise from integrated diagnostic models that combine metabolic, molecular, radiologic, and clinical information rather than prioritizing a single biomarker class.

Several barriers continue to impede clinical translation. Standardized analytical protocols remain lacking, external validation studies are limited, and regulatory pathways for multi analyte metabolomic assays remain poorly defined [127]. Furthermore, implementation challenges related to laboratory infrastructure, assay costs, turnaround time, reimbursement policies, and healthcare accessibility must be addressed before widespread adoption can be considered. These obstacles highlight a broader reality in biomarker development: superior analytical performance alone does not guarantee clinical utility.

Future progress will require a shift in focus from biomarker discovery alone toward the systematic resolution of translational barriers. Establishing harmonized analytical workflows, conducting prospective multicenter validation studies, demonstrating clinical utility, and developing clear regulatory frameworks will be essential. The integration of metabolomic profiles with other biomarker classes remains a promising direction, but only if pursued within a framework that prioritizes real world clinical implementation alongside scientific innovation [130, 131].

In conclusion, specific metabolites demonstrate genuine potential to complement CA19‐9 and enhance the early detection of PDAC, but their replacement of established diagnostic pathways remains unlikely in the near future. Success will depend on coordinated efforts across scientific, clinical, regulatory, and healthcare systems to overcome the substantial barriers that have limited progress thus far. Ultimately, metabolomics is most likely to achieve clinical impact as part of an integrated diagnostic ecosystem rather than as a standalone substitute for existing approaches.

## 12 Future Directions

Future research should shift from identifying additional candidate metabolites toward establishing clinically translatable and reproducible diagnostic frameworks. A major priority is the harmonization of pre‐analytical and analytical procedures, including sample collection, storage, processing, instrument calibration, data normalization, and quality control, to improve inter‐laboratory reproducibility and facilitate cross‐study comparisons.

Large prospective multicenter studies involving geographically and ethnically diverse populations are equally essential to validate proposed metabolite signatures and determine their generalizability across different clinical settings. Such investigations should incorporate external validation cohorts and standardized reporting practices to reduce bias and strengthen the evidence base for regulatory approval and clinical implementation.

Advances in computational methods also warrant more rigorous evaluation. Future ML studies should emphasize transparent model development, robust feature selection strategies, independent external validation, and measures to mitigate overfitting and class imbalance. In addition to reporting predictive performance, investigators should prioritize model interpretability and clinical applicability to facilitate adoption in routine practice.

Rather than pursuing metabolomics as a stand‐alone diagnostic solution, future efforts should explore its integration with complementary modalities such as CA19‐9, imaging techniques, ctDNA, and other molecular biomarkers. Multimodal diagnostic frameworks may provide greater robustness and improve early‐stage detection compared with any individual approach alone.

Finally, successful clinical translation will require addressing practical implementation challenges beyond analytical performance. Cost‐effectiveness analyses, laboratory infrastructure requirements, turnaround times, regulatory pathways, reimbursement considerations, and standardized data‐sharing initiatives should become integral components of future research programs. Collectively, these efforts will determine whether metabolomics can evolve from a promising research platform into a clinically deployable tool for precision detection and management of PDAC.

## 13 Conclusion

Metabolomics has emerged as a powerful approach for investigating the metabolic reprogramming that underlies PDAC and has generated considerable interest as a source of novel biomarkers for early detection. The growing body of evidence demonstrates that alterations in amino acid metabolism, glycolysis, lipid metabolism, and microbiome derived metabolites can provide valuable insights into PDAC biology while simultaneously offering opportunities for diagnostic innovation. Advances in analytical technologies, including MS and NMR spectroscopy, together with the integration of ML approaches, have further expanded the capacity to identify complex metabolic signatures associated with disease initiation and progression.

Despite these advances, the translation of metabolomic discoveries into routine clinical practice remains limited. A recurring theme throughout the current literature is the discrepancy between promising biomarker performance reported in discovery cohorts and the lack of successful implementation in real world clinical settings. Methodological heterogeneity, biological variability, insufficient external validation, interlaboratory reproducibility issues, and the absence of standardized preprocessing and analytical workflows continue to hinder clinical adoption. Furthermore, many proposed biomarkers have been evaluated primarily in retrospective studies and remain insufficiently validated in large prospective multicenter cohorts.

Importantly, current evidence does not support the view that metabolomics will replace established diagnostic modalities in the foreseeable future. Although several metabolite panels have demonstrated diagnostic performance that rivals or exceeds CA19‐9 in selected study populations, the most clinically meaningful advances are likely to arise from multimodal approaches that integrate metabolomics with established serum biomarkers, imaging techniques, molecular diagnostics, and clinical assessment. Recent metabolomic studies have further supported this complementary rather than replacement‐based paradigm. Likewise, emerging metabolic biomarkers such as lipid related signatures continue to provide valuable biological information but require rigorous validation before clinical implementation can be justified.

The integration of artificial intelligence and ML offers substantial opportunities to enhance biomarker discovery and diagnostic accuracy. However, algorithmic sophistication cannot compensate for limitations in study design, cohort size, or data quality. Future ML applications must therefore emphasize transparency, interpretability, external validation, and reproducibility to ensure meaningful clinical utility.

Looking forward, progress in the field will depend less on identifying additional candidate metabolites and more on overcoming the translational barriers that currently limit implementation. Standardized analytical protocols, harmonized reporting frameworks, prospective multicenter validation studies, cost effectiveness analyses, and clearly defined regulatory pathways will be essential for successful clinical adoption. In parallel, a deeper understanding of metabolic heterogeneity across PDAC subtypes and disease stages will be necessary to support personalized diagnostic and therapeutic strategies.

In conclusion, metabolomics has significantly advanced our understanding of PDAC biology and holds genuine promise for improving early detection and patient stratification. Nevertheless, its greatest clinical impact is likely to emerge not as a standalone diagnostic solution, but as an integral component of a comprehensive, multimodal precision oncology framework. Realizing this potential will require coordinated efforts that bridge technological innovation, biological insight, clinical validation, and healthcare implementation.

## Author Contributions

**Javad Yasbolaghi Sharahi:** conceptualization, supervision, writing – original draft, writing – review and editing. **Mohammad Javad Roustaye Gourabi:** investigation, writing – original draft, writing – review and editing. **Mehrangiz Ghafari:** investigation, writing – review and editing, visualization. **Anita Nikoo:** investigation, writing – review and editing. **Yalda Yarahmadi Saki:** investigation, writing – review and editing. **Bita Khanbabaei:** investigation, writing – review and editing. **Masoumeh Khanizadeh:** writing – review and editing, visualization. **Masoud Kargar:** investigation, visualization. **Ali Hashemi:** conceptualization, writing – original draft.

## Funding

The authors have nothing to report.

## Disclosure

All authors have read and approved the final version of the manuscript. The corresponding author (Javad Yasbolaghi Sharahi) had full access to all the data included in this study and takes complete responsibility for the integrity of the data and the accuracy of the data analysis.

## Conflicts of Interest

The authors declare no conflicts of interest.

## Transparency Statement

The corresponding author (Javad Yasbolaghi Sharahi) affirms that this manuscript is an honest, accurate, and transparent account of the study being reported; that no important aspects of the study have been omitted; and that any discrepancies from the study as planned have been explained.

## Data Availability

All data generated and analyzed during the current study are available from the corresponding author on reasonable request.
